# Serum levels of S100B in patients with chronic schizophrenia during treatment augmentation with sarcosine: results of the double-blind, randomized, placebo-controlled PULSAR study

**DOI:** 10.3389/fphar.2026.1705310

**Published:** 2026-03-18

**Authors:** Agnieszka Pawlak, Adam Wysokiński, Dominik Strzelecki

**Affiliations:** 1 Department of Affective and Psychotic Disorders, Medical University of Łódź, Łódź, Poland; 2 Department of Old Age Psychiatry and Psychotic Disorders, Medical University of Łódź, Łódź, Poland

**Keywords:** affective symptoms, negative symptoms, S100B, sarcosine, schizophrenia

## Abstract

**Introduction:**

Sarcosine (N-methylglycine) normalizes glutamatergic neurotransmission in schizophrenia and ameliorates primary negative symptoms. This amino acid may also directly or indirectly influence glial function and therefore levels of S100B, calcium-binding protein linked with glial pathology.

**Aim:**

Investigating an association between initial S100B serum concentrations as a glial marker, its changes, and symptoms severity during use of sarcosine in patients with predominant negative symptoms and stable antipsychotic treatment.

**Methods:**

Sixty subjects with a diagnosis of schizophrenia with predominant negative symptoms completed a 6-month randomized, double-blinded, placebo-controlled prospective study. Participants were randomly assigned in 1:1 ratio and received 2 g of sarcosine or placebo daily *per os*. S100B serum concentrations and severity of symptoms assessments were done in parallel at the beginning, after 6 weeks, and after 6 months of the study. Finally, we obtained results from 15 participants in the sarcosine group and 12 in the placebo group after 6 weeks of receiving augmentation, and from 26 patients in the sarcosine group and 28 in the control group after 6 months of the study. For the clinical evaluation, we used the Positive and Negative Syndrome Scale (PANSS) and the Calgary Depression Scale for Schizophrenia (CDSS).

**Results:**

At baseline, no differences were observed between the sarcosine and placebo groups in PANSS or CDSS scores (all p > 0.35). Sarcosine augmentation led to significantly greater improvement in total, negative, and general psychopathology PANSS scores compared with placebo (t = 2.88–8.23, all p ≤ 0.006), while improvement in depressive symptoms did not reach significance (p = 0.10). Serum S100B levels did not differ between groups at any time point (all p ≥ 0.14; d = −0.06 to −0.40). Mixed-effects ANOVA showed a significant effect of visit on S100B (F (2,52) = 6.10, p = 0.005, η^2^ = 0.12), with no group or interaction effects. Exploratory analyses suggested heterogeneous S100B trajectories across affective outcome subgroups (H = 8.54–9.20, p = 0.03–0.04); however, adjusted regression models did not confirm independent associations with changes in PANSS or CDSS scores.

**Conclusion:**

Sarcosine does not significantly affect S100B concentrations. S100B may be involved in mechanisms related to the presence of affective symptoms in schizophrenia.

**Clinical Trial Registration:**

Clinicaltrials.gov, identifier NCT01503359.

## 1 Introduction

### 1.1 Schizophrenia and glutamatergic system

Schizophrenia is a heterogeneous and very capacious clinical phenomenon. Besides unresolved questions of its pathophysiology, one of the major problems remains an insufficient efficacy of the current form of therapy, especially towards negative and cognitive symptoms. Only advances in this area will enable patients to function better and improve their overall quality of life (Keefe, 2007; Correll and Schooler, 2020).

Glutamatergic (excitatory) and GABAergic (inhibitory) systems are closely related, and both participate in the pathogenesis of positive, negative, affective, and cognitive symptoms in schizophrenia (Coyle, 1996; Olney et al., 1999; Carlsson et al., 2000). Amino acid sarcosine (N-metylglycine) is an endogenous inhibitor of the glycine transporter type I (GlyT1) on astroglial cells (Gomeza et al., 2003; Shibasaki et al., 2017). By increasing the level of the NMDA (N-methyl-D-aspartate) receptor co-agonist with glycine, D-serine, D-cycloserine, or by blocking GlyT1 inhibitors with sarcosine or bitopertin, the NMDA receptor activity on GABAergic inhibitory interneurons is enhanced, clinically leading to amelioration of schizophrenia symptomatology, as well as negative symptoms that are resistant to current treatment (Millan, 2005; Strzelecki et al., 2015b; Balu, 2016; Hirayasu et al., 2016).

Sarcosine, as a modulator of the NMDA receptor glycine site, has been shown to enhance long-term potentiation (LTP) in experimental models, linking its effects to glutamatergic synaptic plasticity (Martina et al., 2004; Mothet et al., 2015). The critical role of NMDA receptors in the induction and maintenance of LTP is well established across multiple brain regions (Bliss and Collingridge, 1993; Nicoll, 2017). In addition, NMDA receptor–dependent signaling is closely involved in activity-dependent synapse formation and stabilization, supporting a role for glycine-site modulation in synaptogenesis and structural synaptic plasticity (Bliss and Collingridge, 1993; Nicoll, 2017).

In our study, we focused on the clinical effects of sarcosine and potential simultaneous changes in neurophysiological parameters. The PULSAR study is a randomized, double-blind, placebo-controlled trial (RCT) that enrolled patients with the diagnosis of chronic and stable schizophrenia with predominant negative symptoms to receive sarcosine or placebo as an addition to a stable antipsychotic treatment. Negative (blunted affect, alogia, anhedonia, avolition, asociality) and cognitive symptoms are crucial features in schizophrenia, arising, *inter alia*, from decreased dopamine turnover in the prefrontal cortex. Negative and cognitive symptoms, while resistant to antipsychotics, are most responsible for the quality of life and functioning of patients (Bowie et al., 2010; Carbon and Correll, 2014; Maroney, 2022).

### 1.2 S100 calcium binding protein B (S100B) - properties and postulated importance in brain diseases

S100B is an acidic protein with a molecular weight of 10 kDa, encoded by a gene located on chromosome 21q22.3 (Rothermundt et al., 2003). S100B is mainly found in brain tissues - astroglia (at the highest concentrations), oligodendrocytes, ependymal cells, choroid plexus epithelial cells, and rarely in neurons (Yelmo-Cruz et al., 2013). In addition to neural tissues, S100B is found in adipocytes, chondrocytes, lymphocytes, Langerhans cells, bone marrow cells, dendritic cells, Schwann cells of the peripheral nervous system and satellite cells of the dorsal root ganglia (Yelmo-Cruz et al., 2013). The level of S100B protein in serum and cerebrospinal fluid is considered an indicator of glial cell activation (Rothermundt et al., 2003). S100B is released from astrocytes both as a soluble protein via regulated non-classical secretion and within extracellular vesicles, particularly under conditions of glial activation, inflammation, or stress, with both mechanisms contributing to its presence in extracellular fluids and blood (Rothermundt et al., 2003; Van Eldik and Wainwright, 2003; Donato et al., 2012). Notably, when the blood-brain barrier (BBB) is intact, S100B cannot pass through it (18 times higher levels in cerebrospinal fluid than peripherally), but disruption of the BBB, as in stroke, ischemia or inflammatory process, leads to an increase in serum S100B levels (Reiber, 2001). Levels of S100B in serum are used as one of the biochemical markers of the extent and course of ischemic stroke and its over-expression indicates worsening in other neurological illnesses (Michetti et al., 2019). Involvement in regeneration processes (at nanomolar concentrations, by activation of anti-apoptotic factor Bcl-2) or apoptotic processes (at micromolar concentrations, inducing pro-inflammatory cytokines, including TNF-alpha) causes S100B, additionally to its marker function, to arouse interest as a target for therapeutic interventions (Lam et al., 2001; Arcuri et al., 2005; Donato et al., 2009; Michetti et al., 2019). It is postulated that S100B may also be a marker of the severity of depressive symptoms and response on antidepressive treatment (Schroeter et al., 2013; Rajewska-Rager and Pawlaczyk, 2016; Navines et al., 2022). Clinical studies underline high initial S100B levels as a predictor of good antidepressant (Arolt et al., 2003; Ambrée et al., 2015; Navines et al., 2022) and electroconvulsive therapy (Arts et al., 2006; Carlier et al., 2019) response in major depressive disorder, while one study indicates the opposite (Jha et al., 2019). The study results show mixed - stable (Ambrée et al., 2015; Navines et al., 2022) or increasing (Arolt et al., 2003) results regarding S100B concentrations after effective antidepressant treatment.

### 1.3 S100B in schizophrenia

S100B is one of the postulated markers observed in the context of schizophrenia symptomatic profile, illness progression, and monitoring of treatment efficacy; however, implementation into practical use is currently at an early stage and requires further research.

Elevated levels of S100B have been consistently reported in both the serum and cerebrospinal fluid of patients with schizophrenia (Steiner et al., 2006; Bernstein et al., 2009; Zhang et al., 2010; Morera-Fumero et al., 2023). This increase likely reflects reactive astrogliosis, a process characterized by increased astrocyte proliferation and metabolic activity in response to neuroinflammatory and excitotoxic stimuli. Histopathological studies have shown increased levels of astrogliosis in approximately 70% of postmortem brains from patients with schizophrenia. These studies highlight morphological changes, including astrocyte hypertrophy and increased S100B levels (Tarasov et al., 2020). Glial cell reactivity contributes to impaired glutamatergic neurotransmission, impaired synaptic regulation, and subsequent cognitive and affective deficits typical of this disorder (Chen et al., 2017; Tarasov et al., 2020). In addition to astrogliosis, in patients with the paranoid form, the white matter adjacent to the dorsolateral prefrontal cortex contained more S100B-positive glial cells, primarily oligodendrocytes, compared to individuals with chronic schizophrenia (Steiner et al., 2008). Rothermund et al. reported an increase in serum S100B levels during the untreated acute episode; however, persistently elevated S100B levels after 6 weeks of treatment were associated with persistent negative symptomatology, which is particularly important for the group we investigate (Rothermundt et al., 2001). Other studies confirm an increase in S100B in an acute episode of untreated schizophrenia (Wiesmann et al., 1999; Lara et al., 2001). One study indicated increase of S100B also in patients with chronic psychosis (Ryoun Kim et al., 2007). The direction of change, i.e., a decrease in S100B concentrations as the disease progresses into a chronic state, was confirmed in one study (Gattaz et al., 2000). Based on these results and our observations from MRI spectroscopy, where glial activity parameters Glx/Cho and Glx/Cr (Glx–a complex of glutamate, glutamine and, partially, GABA; Cho–choline; Cr–creatine) decreased during sarcosine supplementation (Strzelecki et al., 2015a), We hypothesize that a reduction in the severity of sarcosine-induced symptoms may lead to a decrease or stabilization of serum S100B concentrations. Direct evidence linking glycine or sarcosine to specific astrocytic biomarkers such as S100B remains scarce and does not allow for the formulation of clear hypotheses. However, by inhibiting GlyT1, sarcosine increases extracellular glycine levels, indirectly modulating astrocytic calcium signaling, glutamate uptake, and gliotransmitter release, which alters astrocytic reactivity, glutamatergic neurotransmission, and S100B levels. However, due to concentration measurements in peripheral blood it should be emphasized that due to reduced inflammatory processes as part of the treatment, a decrease in BBB permeability may also significantly reduce S100B concentrations in peripheral blood, which can have a fundamental impact on our considerations and conclusions. Our previous studies in this population indicate significant changes in symptomatology (negative symptoms, general psychopathology, and the Positive and Negative Syndrome Scale (PANSS) total score) induced by sarcosine, with concomitant beneficial changes in neuronal viability and neurotransmission in the left hippocampus, left dorsolateral prefrontal cortex, and left frontal white matter (Strzelecki et al., 2015b; Strzelecki et al., 2015a; Strzelecki et al., 2015c), as well as in glial parameters using magnetic resonance spectroscopy compared with placebo (Strzelecki et al., 2015a). In line with these data, we hypothesize that the changes in clinical status and glial parameters observed in the sarcosine group may also be related to or influenced by S100B levels.

To the best of our knowledge, this is the first study to examine a similar combination of these parameters in people diagnosed with schizophrenia.

Our primary objective was to see whether enhancing antipsychotic treatment with sarcosine affects serum S100B levels. A secondary aim of the study was to assess whether S100B levels are correlated with symptom severity and clinical improvement as assessed by the Positive and Negative Syndrome Scale (PANSS) and the Calgary Depression Scale for Schizophrenia (CDSS).

## 2 Materials and methods

### 2.1 Participants and study design

All eligible patients were Caucasian Europeans aged 18–60 with a diagnosis of paranoid schizophrenia (295.30, according to DSM-IV, F20.0 according to ICD-10). We recruited participants at a hospital outpatient clinic. At the first visit, after signing an informed consent, all included patients underwent a structured interview according to ICD-10 and DSM-IV criteria for schizophrenia. The main clinical inclusion criteria were a stable (chronic) mental state with predominant negative symptoms (a minimum of 3 points on each item in the PANSS negative symptoms subscale and a maximum of 3 points on each item in the positive symptoms subscale) and stable treatment for at least 3 months before inclusion in the study. Psychotic exacerbations, suicidal risk, or clozapine treatment were the main exclusion criteria. In some studies, clozapine combined with glutamatergic drugs worsened mental status by increasing the severity of both positive and negative symptoms (Goff et al., 1999; Potkin et al., 1999). We purposefully recruited patients without signs of acute psychosis to focus on primary negative symptoms.

We designed the PULSAR (PoLish Study of SARcosine in Schizophrenia) trial as a 6-month, randomized, double-blinded, placebo-controlled, parallel-group study. All participants were randomly assigned to sarcosine or placebo subgroups in a 1:1 ratio. Sarcosine and a placebo were added to ongoing stable antipsychotic treatment (Supplementary File S1).

Patients received sarcosine and placebo in plastic Eppendorf tubes containing 2 g of the amino acid or microcrystalline cellulose (placebo) and instructions to open the plastic tube, dissolve all the powder in water, and drink once daily in the morning.

**FIGURE 1:**
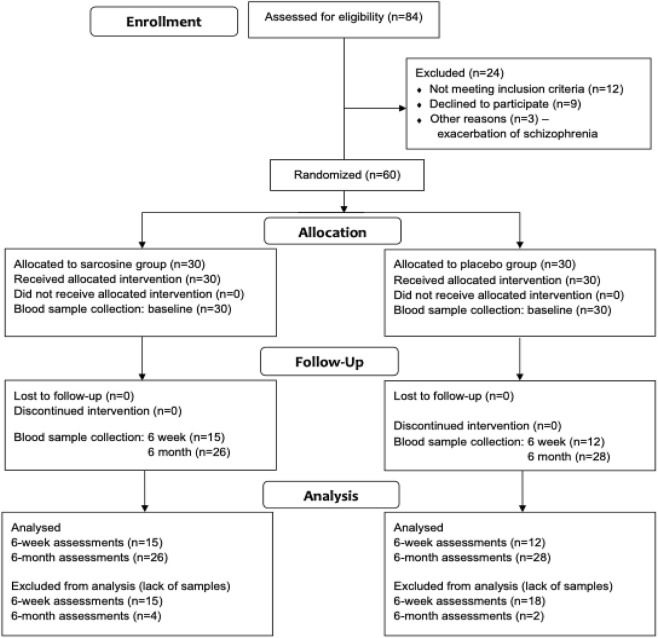
CONSORT diagram for the PULSAR study and blood (serum S100B) assessments. Reasons for missing samples: Week 6 - sarcosine group (15 missing): 11 subjects did not consent to sample collection at this time point, 4 - results below the test sensitivity threshold; placebo group (18 missing): 12 subjects did not consent to sample collection at this time point, 4 - results below the test sensitivity threshold, 2 - sample damage. Month 6 - sarcosine group (4 missing): 4 - results below the test sensitivity threshold; placebo group (2 missing): 1 subject did not consent to sample collection at this time point, 1 - results below the test sensitivity threshold.

In enrollment process sixty subjects were randomized to receive either sarcosine (n = 30) or placebo (n = 30) and completed a 6-month, double-blind, placebo-controlled study. After collecting all the samples, we had S100B levels from 26 patients in the sarcosine group and 28 in the control group at baseline and at the end of the study, which we could use for statistical analysis. For some patients in both groups (15 in sarcosine and 12 in placebo group), we also had protein concentration results at week 6 of augmentation (Figure 1. Consort diagram).

All participants remained on their antipsychotic treatment throughout the study. The two groups were comparable in terms of demographic, clinical (PANSS, CDSS), therapeutic, anthropometric, and metabolic characteristics, except for the percentage of smokers (Table 1).

**TABLE 1:** Baseline participant characteristics.

Parameter	Participants		p
	Sarcosine (n = 30)	Placebo (n = 30)
Men	20 (66.7%)	15 (50.0%)	NS
Age [years]	36.9 ± 11.4 [32.6–41.1]	40.2 ± 10.1 [36.4–43.9]	NS
Smoking	10 (33.3%)	19 (63.3%)	0.02
Cardio-metabolic characteristics			
SBP [mm Hg]	124.3 ± 16.1 [118.3–130.3]	126.7 ± 16.4 [120.5–132.8]	NS
DBP [mm Hg]	75.0 ± 9.3 [71.5–78.4]	79.3 ± 9.2 [75.8–82.7]	NS
TC [mg/dL]	200.4 ± 32.2 [188.3–212.4]	221.2 ± 54.1 [201.0–241.4]	NS
HDL [mg/dL]	45.9 ± 18.4 [39.1–52.8]	45.5 ± 14.7 [40.0–51.0]	NS
LDL [mg/dL]	122.2 ± 30.1 [111.0–133.5]	143.6 ± 43.8 [127.2–160.0]	NS
TGA [mg/dL]	159.8 ± 87.7 [127.1–192.5]	161.2 ± 106.7 [121.4–201.1]	NS
FPG [mg/dL]	96.4 ± 13.8 [91.2–101.6]	97.5 ± 22.8 [89.0–106.1]	NS
TSH [μIU/mL]	1.7 ± 0.9 [1.4–2.1]	1.5 ± 0.6 [1.2–1.7]	NS
PRL [ng/mL]	32.8 ± 30.5 [21.4–44.2]	31.5 ± 36.3 [17.9–45.1]	NS
Antihypertensive treatment	4 (13.3%)	7 (23.3%)	NS
Lipid-lowering treatment	1 (3.3%)	2 (6.7%)	NS
Antidiabetic treatment	1 (3.3%)	0	NS
Metabolic syndrome	14 (46.7%)	18 (60.0%)	NS
Dyslipidemia	23 (76.7%)	25 (83.3%)	NS
Impaired fasting glucose	8 (26.7%)	9 (30.0%)	NS
Clinical characteristics			
Treatment duration [years]	13.9 ± 8.9 [10.6–17.3]	11.6 ± 4.9 [9.7–13.4]	NS
Number of hospitalizations	4.7 ± 5.6 [2.6–6.8]	4.1 ± 4.8 [2.3–5.9]	NS
Time from last hospitalization [years]	2.9 ± 4.0 [1.4–4.5]	4.7 ± 4.6 [2.9–6.5]	NS
Number of APs 1 2 3	13 (43.3%) 16 (53.3%) 1 (3.3%)	11 (36.7%) 17 (56.7%) 2 (6.7%)	NS
SGAs	18 (60.0%)	19 (63.3%)	NS
FGAs	2 (6.7%)	8 (26.7%)	NS
Antidepressants	14 (46.7%)	11 (36.7%)	NS
Initial PANSS score	69.4 ± 13.7 [64.3–74.6]	72.4 ± 12.4 [67.8–77.1]	NS
Positive subscale	10.0 ± 2.9 [8.9–11.1]	10.4 ± 3.1 [9.2–11.5]	NS
Negative subscale	25.4 ± 5.2 [23.5–27.4]	26.1 ± 5.0 [24.2–28.0]	NS
General psychopathology subscale	34.0 ± 8.1 [30.9–37.0]	35.9 ± 7.4 [33.1–38.7]	NS
Initial CDSS score	3.7 ± 3.0 [2.6–4.8]	3.6 ± 2.7 [2.5–4.6]	NS
Patients with depression	6 (20.0%)	5 (16.7%)	NS
Weight [kg]	91.8 ± 23.7 [82.7–100.8]	86.4 ± 16.3 [80.3–92.5]	NS
BMI [kg/m^2^]	34.1 ± 21.9 [25.8–42.5]	29.4 ± 4.9 [27.6–31.3]	NS

Data given as: n (%) or mean ± standard deviation [95% CI].

SBP, systolic blood pressure; DBP, diastolic blood pressure; TC, total cholesterol; HDL, high-density lipoproteins; LDL, low-density lipoproteins; TGA, triglycerides; FPG, fasting plasma glucose; TSH, thyroid-stimulating hormone; PRL, prolactin; APs, antipsychotics; SGAs, second-generation antipsychotics; FGAs, first-generation antipsychotics; PANSS, Positive and Negative Syndrome Scale; CDSS, Calgary Depression Scale for Schizophrenia; BMI, body mass index.

All participants were thoroughly informed of the study’s objectives and methods and gave written informed consent to participate in the study. The Bioethics Committee of the Medical University of Lodz approved the study protocol (permission number and date: RNN/153/08/KE, 15.07.2008). There was no financial involvement from the industry. The authors were supported by Polish Ministry of Science and Higher Education (grant N402 268836). For more information on the PULSAR study, visit ClinicalTrials.gov, study identifier: NCT01503359.

### 2.2 Measurements

All blood measurements were taken at least twice - at the visit before the implementation of sarcosine or placebo and at the last study visit after 6 months. In 15 in the sarcosine group and 12 patients in the placebo group, we performed additional S100B assessments after 6 weeks.

#### 2.2.1 Clinical assessments

We used the PANSS scale and its subscales of positive, negative, and general psychopathology symptoms to assess schizophrenia symptomatology, while we applied the Calgary Depression Scale for Schizophrenia (CDSS) to assess depression severity (Kay et al., 1987; Addington et al., 1990). One trained assessor performed all scales on each participant continuously. According to the authors’ instructions, patients were classified as depressed when the CDSS score exceeded 6 points.

#### 2.2.2 Blood

Blood samples were taken after at least 8 hours of overnight fasting between 7:00 and 8:00 am. All samples were immediately transferred to the central laboratory for preparation and analysis. Blood was centrifuged for 10 min at 3,500 rpm at 22 °C. We used Dirui CS-400 equipment (Dirui, China) to analyze serum glucose and lipid levels. Serum S100B concentration was measured according to the manufacturer’s instructions using a commercially available high-sensitivity ELISA kit (Diaclone, Besancon Cedex, France), intra-test CV < 4.5%, inter-test CV < 9.2%. Prior to ELISA procedures, serum samples were frozen at −80 °C. The optical density of the wells was measured at the Central Scientific Laboratory of the Medical University of Lodz using an automated microplate reader (Emax; Molecular Devices, United States).

### 2.3 Statistical analysis

During the PULSAR study, the randomization schedule was not interrupted until all data were collected. Simple descriptive statistics (means, standard deviations, 95% confidence interval [CI]) were generated for all continuous variables, while the number of patients and percentages were assigned to discrete variables. The normality of distribution was assessed using the Shapiro-Wilk test. We used a t-test for variables with a normal distribution, in other circumstances we applied the Wilcoxon rank-sum test. Mixed-effect two-way ANOVA design was used to analyze changes of S100B concentrations between groups (random between-subjects factor) and between visits (fixed within-subjects factor). For the ANOVA the missing records were removed from the analysis. Sphericity was tested and adjusted, if necessary. We used post-hoc pairwise tests with Bonferroni adjustment for p-values. Differences between proportions were analyzed using the Fisher’s exact test, while intergroup differences were assessed using the t-test. Associations between S100B levels and symptom severity were examined using Spearman’s rank correlation coefficient and linear regression (controlled for age, gender, smoking, and BMI). Appropriate effect size measures (Cohen’s d and generalized η^2^) are reported. The significance level was set at p < 0.05 (two-sided).

## 3 Results

At the baseline, there were no significant differences in PANSS (t (58) = 0.89, p = 0.37) or CDSS (t (58) = -0.13, p = 0.89) scores between groups, see Table 1. Consistent with the overall clinical results, patients in the sarcosine group showed significantly greater improvement in total (t (56.96) = 4.8124, p < 0.001), negative (t (42.62) = 8.23, p < 0.001), and general psychopathology (t (56.72) = 2.88, p = 0.006) PANSS scores compared with the placebo group. Changes in depressive symptoms measured by the CDSS also favored sarcosine over placebo, but the difference was not significant (t (45.17) = 1.65, p = 0.10).

**FIGURE 2:**
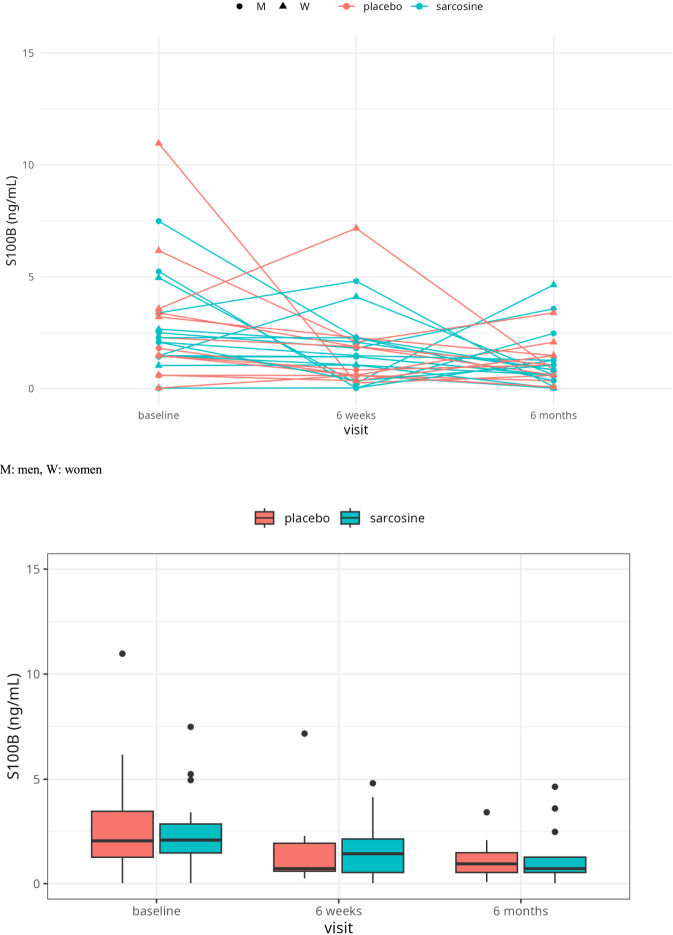
Levels of S100B during the study. M: men, W: women.

Figure 2 presents serum S100B levels across the three study time points (baseline, 6 weeks, and post-treatment). At baseline, there were no differences between the study groups (sarcosine: 15.5 ± 44.7 ng/mL, placebo: 9.8 ± 17.6 ng/mL, t (54) = -0.642, p = 0.52, Cohen’s d = −0.17). After 6 weeks, no significant differences were observed (sarcosine: 1.56 ± 1.36 ng/mL, placebo: 1.47 ± 1.87 ng/mL, t (27) = -0.156, p = 0.88, Cohen’s d = −0.06). At the end of the study, mean S100B concentrations remained comparable (sarcosine: 6.8 ± 19.9 ng/mL, placebo: 1.2 ± 1.6 ng/mL, t (54) = -1.51, p = 0.14, Cohen’s d = −0.40).

Using the mixed-effects two-way ANOVA analysis we have found a significant effect of the visit (F (2,52) = 6.10, p = 0.005, corrected for sphericity). The effect size was small (generalized η^2^ = 0.12). There were no significant effects of the study group (F (1, 26) = 0.09, p = 0.76, generalized η^2^ < 0.01) and the group:visit interaction (F (2,52) = 0.18, p = 0.83, generalized η^2^ < 0.01). Further post-hoc pairwise tests with Bonferroni-adjusted p values did not show any significant differences for any combination of visit in the placebo or sarcosine groups. See Figure 2 for details.

**FIGURE 3:**
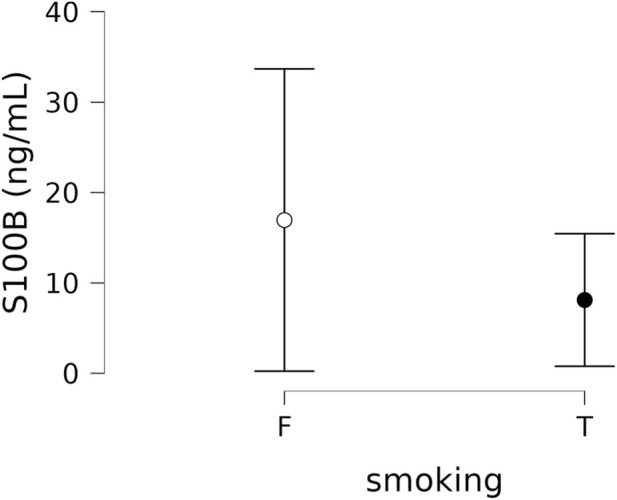
Baseline S100B levels in smokers and non-smokers. Graph shows means and 95% confidence intervals. F: false, T: true.

As there were significantly more smokers in the placebo group at the baseline, we tested whether the baseline difference in S100B concentrations between smokers (n = 28, 8.11 ± 18.93 ng/mL) and non-smokers (n = 28, 16.95 ± 43.12 ng/mL) was significant. We found the difference to be not significant (t (df = 54) = 0.99, p = 0.32), see Figure 3.

**FIGURE 4:**
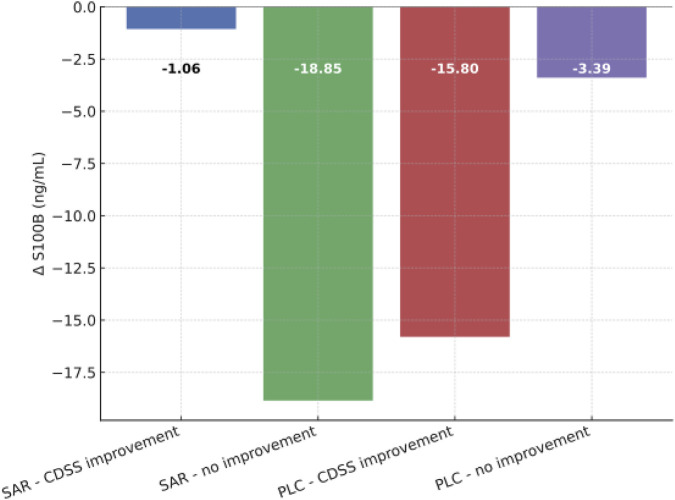
Changes in S100B according to CDSS improvement status. SAR, sarcosine group; PLC, placebo group; CDSS, Calgary Depression Scale for Schizophrenia; ΔS100B, serum levels difference between the end and the beginning of treatment augmentation with sarcosine or placebo.

Exploratory analyses considering affective symptomatology showed that changes in S100B were associated with depressive outcomes, but these results are not consistent. In the sarcosine group, patients with CDSS improvement (CDSS score decreased to 6 points or below) displayed only minimal reductions in S100B (mean reduction −1.06 ng/mL), whereas those without improvement showed larger decreases (Figure 4). In the placebo group, depressive improvement was associated with a marked decrease in S100B, while non-improvers had minimal changes. Kruskal–Wallis test revealed significant differences in S100B changes across all four subgroups defined by CDSS improvement (H = 8.54, df = 3, p = 0.04) and worsening (H = 9.20, df = 3, p = 0.03, defined as obtaining more than 6 points in the CDSS). These results indicate heterogeneity in S100B trajectories depending on treatment group and affective outcome. Finally, linear regression analyses adjusted for age, sex, smoking, and BMI did not reveal significant independent associations of S100B with changes in the PANSS (adj. R^2^ = −0.04, F (4,50) = 0.49, p = 0.75) or CDSS (adj. R^2^ = −0.04, F (4, 50) = 0.45, p = 0.77) scores.

## 4 Discussion

In our study, we examined the role of the glial protein S100B in patients with schizophrenia and its relationship with psychopathological symptoms including affective dimension. S100B, primarily produced by astrocytes, is increasingly recognized as a biomarker reflecting glial dysfunction, neuroinflammation, and BBB alterations. Elevated serum S100B levels have been consistently reported in schizophrenia, particularly during exacerbations, and have been also linked to negative and general psychopathology symptoms as assessed by the PANSS (Rothermundt et al., 2001; Steiner et al., 2008; Aleksovska et al., 2014; Morera-Fumero et al., 2023). Changes in S100B concentrations in schizophrenia require differential interpretation depending on directionality and clinical context. Increases in S100B are generally interpreted as markers of astrocytic activation, neuroinflammatory processes, or BBB dysfunction, reflecting pathological or stress-related glial states. In contrast, decreases in S100B may indicate attenuation of reactive gliosis, reduced inflammatory signaling, or restoration of BBB integrity, particularly in association with clinical improvement. It is worth noting that alterations in BBB integrity also influence extracellular neurotransmitter homeostasis, particularly glutamate dynamics, with BBB disruption permitting greater passage of excitatory molecules into brain tissue, thereby altering excitation–inhibition balance at the network level (Boyko et al., 2023; Friedman et al., 2025). Such changes in the neurochemical environment may contribute to dissociations between peripheral glial biomarkers and clinical symptom trajectories. In this context, it is worth noting that sarcosine may exert effects beyond neuronal NMDA receptor modulation, as glycine transporter inhibition can influence astrocytic and endothelial function, and glycine itself has documented anti-inflammatory properties, including modulation of pro-inflammatory signaling pathways such as NF-κB (Aguayo-Cerón et al., 2023). These non-neuronal actions may partially uncouple biomarker dynamics from symptomatic outcomes, offering a plausible explanation for heterogeneous or negative associations observed in glutamatergic intervention studies.

We did not observe significant differences between sarcosine and placebo groups in absolute changes of S100B in our study group; however, in the placebo group a significant decrease was found between baseline and post-treatment assessment. The decrease in concentration in the placebo group may indicate a progressive decline in astroglial activity in these patients. The simultaneous lack of a decrease in S100B concentration in the sarcosine group, which improved in the PANSS, may reflect stabilization of glial cell activity, what may support our findings from MRI part of PULSAR project (Strzelecki et al., 2015a). However, these results may be also an artifact.

Previous studies have demonstrated associations between S100B levels and affective symptomatology, suggesting a broader role of this astrocyte-derived protein in mood dysregulation. Elevated serum S100B has been reported in mood disorders and has been shown to decrease following effective antidepressant treatment, with higher baseline levels associated with a greater likelihood of treatment response and more favorable longitudinal outcomes (Jang et al., 2008; Schroeter and Steiner, 2009; Ambrée et al., 2015). In line with these observations, our findings revealed associations between S100B concentrations and affective symptom severity, as measured by CDSS and mood PANSS domain, but exclusively in the placebo group. In contrast, under sarcosine treatment, changes in S100B were not consistently related to depressive symptom improvement, and pronounced reductions in S100B were observed also in patients without clinical response. This pattern suggests that glutamatergic modulation may alter or attenuate the coupling between astrocytic S100B signaling and the expression of affective symptoms. Consequently, changes in circulating S100B may reflect modulation of glial or inflammatory processes rather than direct changes in psychotic or affective symptom severity. Decreases in S100B accompanied by affective improvement in the placebo group may therefore indicate a downregulation of astroglial reactivity, whereas relatively stable S100B levels during sarcosine treatment may reflect ongoing astrocytic involvement in glutamatergic neurotransmission, potentially contributing to clinical effects through mechanisms not directly captured by serum S100B dynamics. Taken together, these findings suggest that S100B may represent a state-sensitive but context-dependent marker of affective and overall psychopathological symptomatology in schizophrenia. However, its utility as a standalone biomarker of treatment response appears limited, and its interpretation likely requires consideration of concurrent neurochemical modulation and glial activity.

Several limitations of our study should be acknowledged. First, the sample size was relatively modest, which may have limited the statistical power to detect smaller effects and increased the risk of type II errors. Second, the study design did not include a healthy control group, making it difficult to establish whether the observed S100B dynamics are specific to schizophrenia or reflect more general mechanisms of psychiatric illness. Third, S100B was measured only peripherally in serum, which may not fully capture central nervous system processes, particularly given the possible influence of BBB integrity. Fourth, several potential confounders such as smoking status, type and dose of antipsychotic medication, and comorbid affective symptoms were only partially controlled for in subgroup analyses, which may have introduced bias. Fifth, the follow-up period was limited to 6 months, restricting our ability to evaluate long-term trajectories of S100B and their relationship with chronic symptom dimensions. Finally, as this was an exploratory study, findings should be interpreted with caution and require replication in more homogenously treated, larger, longitudinal cohorts with multimodal biomarker assessments.

In summary, our findings highlight the relevance of the glial protein S100B as a potential biomarker of both affective and global psychopathological symptoms in schizophrenia. Although no consistent differences between treatment groups were observed, S100B dynamics were significantly associated with changes in depressive symptoms as measured by CDSS and correlated with PANSS domains, particularly in the placebo group on the end of active phase of the study. These results support the hypothesis that S100B allows monitoring the severity of schizophrenia symptoms, including depressive symptomatology, as it was noted in the control group. The effect of glutamatergic modulation distinguished both groups in our study with respect to changes in peripheral S100B concentrations. While its role as a treatment-specific biomarker remains uncertain, S100B appears to capture clinically meaningful changes in symptomatology and may therefore have potential utility for monitoring disease course. Future research in larger, longitudinal cohorts is needed to validate its predictive potential and clarify whether interventions targeting astroglial activity could provide therapeutic benefit.

## Data Availability

The raw data supporting the conclusions of this article will be made available by the authors, without undue reservation.
