# Supplementary material for: Serum levels of S100B in patients with chronic schizophrenia during treatment augmentation with sarcosine: results of the double-blind, randomized, placebo-controlled PULSAR study
Source: Front Pharmacol. 2026 Mar 18;17:1705310. doi: 10.3389/fphar.2026.1705310 (PMC13038986; doi:10.3389/fphar.2026.1705310)
Supplement: Supplementary file 1 [file Supplementaryfile1.docx]

Supplementary file 1. Antipsychotic and antidepressive drugs in study group.

| Sarcosine group | | | Placebo group | | |
| --- | --- | --- | --- | --- | --- |
| Patient | Antipsychotic  *(dose, mg)* | Antidepressant  *(dose, mg)* | Patient | Antipsychotic  *(dose, mg)* | Antidepressant  *(dose, mg)* |
| 1 | Ari *(7.5)* | Ser *(50)* | 1 | Ola *(10)* |  |
| 2 | Ari *(30)* | Ser *(25)* | 2 | Ola *(20)* |  |
| 3 | Ari *(15)*, Ola *(20)* |  | 3 | Ola *(20)* |  |
| 4 | Ari (30), Ola *(5)* |  | 4 | Ari *(30)* |  |
| 5 | Ola *(10)* | Ser *(50)* | 5 | Ari *(30)* |  |
| 6 | Ola *(2.5)* | Ser *(50)* | 6 | Ola *(5)* | Ser *(50)* |
| 7 | Ami *(600)*, Que *(25)* |  | 7 | Ola *(20)* | Ser *(200)* |
| 8 | Ris *(4)* | Cit *(30)* | 8 | Ami *(200)*, Que *(150)* |  |
| 9 | Ris *(5.5)* | Cit *(20)* | 9 | Ami *(400)*, Que *(100)* |  |
| 10 | Ola *(10)* |  | 10 | Ari *(30)*, Que *(100)* |  |
| 11 | Que *(800)* |  | 11 | Ari *(30)*, Que *(25)* |  |
| 12 | Sul *(500)* |  | 12 | Flp *(200/3 weeks*)* |  |
| 13 | Per *(300)* |  | 13 | Ami *(400)*, Ola *(20)* |  |
| 14 | Ami *(600)*, Ola *(20)* |  | 14 | Ari *(7.5)*, Ola *(12.5)* |  |
| 15 | Ola *(10*), Ris *(3)* |  | 15 | Flp *(3)*, Ola *(20)* |  |
| 16 | Ola *(10)*, Sul *(400)* |  | 16 | Per *(125)*,  Ris *(50/2 weeks*)* |  |
| 17 | Ola *(25)* | Esc *(10)* | 17 | Que *(200)*, Ris *(4)* |  |
| 18 | Que *(400)*,  Ris *(50/2 weeks*)* |  | 18 | Que *(700)*,  Zuc *(300/2 weeks*)* |  |
| 19 | Ari *(30)*, Flp *(6)* |  | 19 | Ami *(200)* | Ven *(225)* |
| 20 | Ami *(600)*, Pro *(300)* |  | 20 | Flp *(200/4 weeks*)*, Ris *(0.5)* |  |
| 21 | Ami *(300)* | Fvx *(100)* | 21 | Sul *(50)* | Cit *(60)* |
| 22 | Flp *(6)*, Per *(300)* |  | 22 | Flp *(3)* | Cit *(20)* |
| 23 | Ami *(800)*, Ola *(20),* Que *(200)* |  | 23 | Ami *(600)*, Ola *(10)* | Cit *(10)* |
| 24 | Ami *(400)*, Ola *(5)* | Flx *(10)* | 24 | Ari *(30)*, Que *(600)* | Cit *(20)* |
| 25 | Ari *(30)*, Ola *(15)* | Ser *(100)* | 25 | Ami *(400)*, Hal *(3)*, Que *(400)* |  |
| 26 | Ola *(15)*, Ami *(200)* | Ser *(200)* | 26 | Lev *(50)*, Que *(200)* | Ser *(50)* |
|  |  |  | 27 | Ari *(30)*, Ris *(2.5)* | Fvx *(50)* |
|  |  |  | 28 | Ami *(600)*, Pro *(100)* | Ven *(225)* |

Abbreviations

Antipsychotics: Ami – amisulpride, Ari – aripiprazole, Flp – flupenthixol, Hal – haloperidol, Lev – levomepromazine, Ola - olanzapine, Per – perazine, Pro – promazine, Que - quetiapine, Ris – risperidone, Sul – sulpiride, Zip – ziprasidone, Zuc – zuclopenthixol, * - long-acting injection.

Antidepressants: Cit – citalopram, Clo – clomipramine, Esc – escitalopram, Flx – fluoxetine, Fvx – fluvoxamine, Ser – sertraline, Ven – venlafaxine.
